# Intelligent Fault Diagnosis of Machinery Using BPSO-Optimized Ensemble Filters and an Improved Sparse Representation Classifier

**DOI:** 10.3390/s25165175

**Published:** 2025-08-20

**Authors:** Yuyao Tang, Yapeng Yang, Xiaoyu Zhao, Qi Lv, Jiapeng He, Zhiqiang Zhang

**Affiliations:** 1 China Institute for Radiation Protection, Taiyuan 030006, China; 2 School of Electrical Engineering and Automation, Hefei University of Technology, Hefei 230009, China

**Keywords:** intelligent fault diagnosis, feature selection, sparse representation classifier, binary particle swarm optimization, cumulative reconstruction residual

## Abstract

In this paper, we propose an ensemble approach for the intelligent fault diagnosis of machinery, which consists of six feature selection methods and classifiers. In the proposed approach, six filters, based on distinct metrics, are utilized. Each filter is combined with an improved sparse representation classifier (ISRC) to form a base model, in which the ISRC is an improved version of a sparse representation classifier and has the advantages of high classification accuracy and being less time consuming than the unimproved version. For each base model, the filter selects a feature subset that is used to train and test the ISRC, where the two hyper-parameters involved in the filter and ISRC are optimized by the binary particle swarm optimization algorithm. The outputs of six base models are aggregated through the cumulative reconstruction residual (CRR), where the CRR is devised to replace the commonly used voting strategy. The effectiveness of the proposed method is verified on six mechanical datasets involving information about bearings and gears. In particular, we conduct a detailed comparison between CRR and voting and carry out an intensive exploration into the question of why CRR is superior to voting in the ensemble model.

## 1. Introduction

Machinery fault diagnosis plays a critical role in ensuring operation reliability, offering guidance for timely mechanical maintenance and preventing unnecessary economic losses. Traditional fault diagnosis methods heavily rely on diagnosticians, who make diagnostic decisions by analyzing the monitored signals; such processes are labor-intensive, and diagnosticians often struggle to promptly process large volumes of monitored signals [1]. Currently, intelligent fault diagnosis approaches are gaining increasing attention, involving the replacement of human diagnosticians with artificial intelligence techniques to rapidly process large amounts of collected signals [2].

The general framework of intelligent fault diagnosis includes data acquisition, feature extraction, feature selection, and fault classification [3]. In a such framework, plenty of mechanical signals, which provide the most intrinsic information about machinery faults, are first acquired. These signals can be collected by the installed sensors, such as accelerometers, or can be simulated according to the principles of mechanical dynamics. As stated in [4], the simulated data can be used to clearly detect failures in physical bearing measurements. Subsequently, the acquired signals are processed to extract representative features, among which the most salient features are then selected. Lastly, the supervised classifier is employed to classify different mechanical faults based on the selected features. Among these stages, feature selection is crucial. This is because the extracted features may have large dimensionality, which may increase the computational costs of a subsequent classifier and degrade the classification accuracy of the classifier [5]. Feature selection helps mitigate these issues by removing irrelevant or redundant features and improve the classification accuracy by retaining the most discriminative features [6].

In general, feature selection methods can be broadly categorized as filters and wrappers [7]. Filter approaches score each feature based on certain evaluation criteria and select the top-ranked features, while wrapper approaches associate selected features with classifiers and select the optimal feature subset that maximizes the classification accuracy of a classifier. Compared to wrapper approaches, filter approaches are more time-efficient, meaning it is more applicable in the field of mechanical fault diagnosis. In earlier research, Lei et al. [5] applied the distance evaluation technique (DET) for the fault diagnosis of a heavy oil catalytic cracking unit, where the diagnostic accuracy increased from 86.18% to 100% after feature selection. Zheng et al. [8] selected five features from twenty original features for identifying rolling bearing faults using the Laplacian score (LS), thus achieving 100% testing accuracy. Recently, Vakharia et al. [9] employed ReliefF for the fault diagnosis of bearings, resulting in an accuracy of 6.45% after feature selection. Li et al. [10] utilized minimal redundancy–maximal relevance (mRMR) to select four sensitive features from twenty features for the fault diagnosis of planetary gearboxes; a diagnostic accuracy of 96.94% was achieved. Additionally, both Wei et al. and Wang et al. applied self-weight (SW) to the fault diagnosis of rolling bearings in [11,12], respectively. A literature review highlights the successful applications of filters in machine fault diagnosis. However, the aforementioned studies share a common limitation: only a single filter (e.g., LS, DET, ReliefF, SW, and mRMR) is adopted. Meanwhile, each filter utilizes a different metric (e.g., entropy, distance, locality, etc.), which may lead to variability on different datasets. To be specific, a filter that performs well on one dataset may perform poorly on another dataset [13].

Ensemble learning holds the potential to overcome the aforementioned weakness, and an ensemble of filters has been investigated in data classification [14]. The generalization capability of ensemble learning is theoretically supported by the statistics, which focus on reducing model complexity and enhancing base learner diversity, thereby minimizing generalization error [15]. In [14], several filters and a specific classifier are adopted. Each filter is integrated with the classifier to create a base model. In this model, the filter selects a unique subset of features used to train and test the classifier. The outputs of all base models are combined using a simple voting strategy. Experimental results consistently demonstrate that the ensemble filters method outperforms each filter in most cases and obtains good performance independently on the dataset. Despite the improved performance achieved by studies using ensemble filters, two drawbacks remain. First, each filter in the ensemble filters selects the same number of features for classification, which is predefined by users. This may fail to give rise to the optimal results because different filters have distinct characteristics; in achieving the same result, one filter may select many features, whereas another may need only a few. Second, the voting strategy has the following three limitations: (1) It is difficult to use the voting strategy to make correct decisions when two classes acquire the same maximum number of votes. (2) It is likely that one will make different decisions when two classes are extremely similar. (3) The voting strategy requires that most base models perform well, but this cannot always be guaranteed.

Moreover, the classifier also plays an indispensable role in an ensemble model aiming to recognize different categories based on the selected features. Over the past few decades, various classifiers have been developed, with the sparse representation classifier (SRC) [16] offering a novel approach to classification. It has been extensively applied in many fields, including face recognition [17], anomaly detection [18], and fault diagnosis [19], among others, and excellent classification accuracies were obtained. Nonetheless, the standard SRC utilizes all training samples to represent the testing sample and solves the representation coefficients through greedy algorithms, which makes it computationally intensive.

Aiming to resolve all above-mentioned problems, this paper proposes an ensemble approach for the intelligent fault diagnosis of machinery. In this approach, six filters are combined with an improved sparse representation classifier (ISRC) to separately form six base models. For each base model, binary particle swarm optimization (BPSO) is employed to optimize its two hyper-parameters related to the filter and the ISRC, respectively. After optimization, each filter selects a feature subset which is then used to train and test the ISRC. Finally, the outputs of six ISRCs are aggregated by the cumulative reconstruction residual (CRR) strategy instead of voting. The effectiveness of the proposed approach is verified using six mechanical datasets on bearings and gears.

In summary, the contributions of this paper are as follows.

(1) To overcome the drawback of the SRC, we propose the ISRC, which inherits the advantage of high classification accuracy in the standard SRC, while being less time consuming.
(2) To overcome the limitations of the voting strategy, CRR is proposed, which is capable of making more accurate and reliable decisions than the commonly used voting approach.
(3) To enhance the generalization ability of the diagnosis model, an ensemble approach is presented by iterating each base model; meanwhile, the BPSO is adopted to optimize two hyper-parameters in each base model to achieve a better performance.

The remainder of this paper is organized as follows. Relevant findings from the literature are briefly reviewed in Section 2. The proposed ISRC is presented in Section 3. Detailed descriptions of the proposed ensemble approach for the intelligent fault diagnosis of machines are provided in Section 4. In Section 5, several comparative experiments are conducted to validate the effectiveness of the proposed approach. Finally, Section 6 concludes this paper.

## 2. Review of Basic Knowledge

### 2.1. Filters

The proposed approach involves six representative and extensively used filters that are based on distinct metrics, each of which is described in detail below.

(1) Laplacian score (LS): The LS is proposed in [20]; it gives each feature a specific score that measures the locality preservation capability of the corresponding feature. Features are ranked based on these scores, with smaller values indicating greater importance.
(2) Self-weight (SW): SW is proposed in [11]; it adaptively evaluates the contribution of each feature without requiring class labels. The main steps of SW include the following: calculate the self-similarity factor; build the weight matrix; calculate the self-weight value of each feature. When carrying out feature selection, the features with large self-weight values are prioritized.
(3) Distance discriminative technique (DDT): The DDT is developed in [21] to identify features with strong class separability and within-class compactness. It is particularly effective for high-dimensional data and requires relatively low computational costs.
(4) Distance evaluation technique (DET): The fundamental rationale of the DET holds that features which minimize intra-class distances and maximize inter-class distances are preferable, and such features tend to be given a higher score.
(5) ReliefF: This is a supervised algorithm which gives features a higher score if they can perform well in discriminating among neighbors of other classes and cluster the neighbors in the same classes. The features corresponding to larger weights are selected, while those with smaller weights are discarded.
(6) Minimal redundancy–maximal relevance (mRMR): mRMR is proposed in [22] to select features with maximum relevance and minimum redundancy; mutual information is typically used to measure feature relevance.

### 2.2. BPSO

BPSO [23] is an extended version of PSO [24], designed for solving integer programming problems. Before describing BPSO, we provide a brief overview of PSO.

The development of PSO was inspired by the behavior of biological populations, where the population (swarm) is composed of a group of particles. Each particle is a potential solution represented by velocity, position, and fitness value. The fitness value, calculated through the fitness function, indicates the quality of the solution. When searching for an optimal solution, the Pbest of each personal particle and the Gbest of all particles must be calculated at each iteration. Based on the current Pbest and Gbest, the speed and position at the next iterations of each particle are updated. Mathematically, we assume that $\{p_{1},$$p_{2},\ldots ,p_{n}\}$ is a swarm including *n* particles, where $p_{i}={\left(p_{i1},p_{i2},\ldots ,p_{id}\right)}^{T}$ is the *i*-th particle searching optimal solution in *d*-dimensional space. The velocity of $p_{i}$ is denoted as $v_{i}={\left(v_{i1},v_{i2},\ldots ,v_{id}\right)}^{T}$, and the corresponding Pbest is referred to as $u_{i}={\left(u_{i1},u_{i2},\ldots ,u_{id}\right)}^{T}$. The Gbest of the swarm is $u_{g}={\left(u_{g1},u_{g2},\ldots ,u_{gd}\right)}^{T}$. Based on these definitions, the updated formulas of velocity and position are

(1) $$\begin{array}{c} v_{ij}^{t+1}=wv_{ij}^{t}+c_{1}r_{1}\left(u_{ij}^{t}-p_{ij}^{t}\right)+c_{2}r_{2}\left(u_{gj}^{t}-p_{id}^{t}\right)\\ p_{ij}^{t+1}=p_{ij}^{t}+v_{ij}^{t+1} \end{array}$$

where i=1,2,…,n, and j=1,2,…,d. *t* is the current iteration number. $c_{1}$ and $c_{2}$ are the acceleration factors, which are non-negative. $r_{1}$ and $r_{2}$ are two random numbers distributed in [0,1]. *w* is the inertia weight to balance the capability of local search and global search, which are updated by $w^{t}=w^{max}-\left(w_{max}-w_{min}\right)\times t/n_{iter}$, where $w_{max}$ and $w_{min}$ denote the maximum and minimum inertia weight. $n_{iter}$ is the maximum iteration number.

BPSO is obtained by modifying PSO in two aspects [7]. First, the velocity, $v_{ij}$, is limited to the interval $\left(0,1\right)$ by means of a sigmoid function, i.e., $v_{ij}^{t+1}=1/\left(1+exp\left(-v_{ij}^{t+1}\right)\right)$. Second, $p_{ij}$, $u_{ij}$, and $u_{gj}$ are explicitly constrained to 0 or 1, where the restriction of $P_{id}$ is achieved by

(2) $$p_{ij}^{t+1}=\left\{\begin{array}{c} 1\;\;,\;\mathrm{if}\;\;rand<sigmoid(v_{ij}^{t+1})\;\;\\ 0\;\;,\;\mathrm{otherwise}\; \end{array}\right.$$

where rand is a rand number distributed in the interval $\left(0,1\right)$.

## 3. The Improved Sparse Representation Classifier

The fundamental rationale of the SRC is to first represent a testing sample as a linear combination of the training samples with sparse representation coefficients. Subsequently, the type of testing sample can be determined by the reconstruction residual.

Suppose $\left\{x_{i}^{c},i=1,2,\ldots ,N_{c};c=1,2,\ldots ,C;\sum_{c=1}^{C}N_{c}=N\right\}$ represents a training set containing *N* samples belonging to *C* categories, where $x_{i}^{c}\in {ℜ}^{D}$ denotes the *i*-th sample of the *c*-th class, and *D* is the dimension. $N_{c}$ is the number of samples in the *c*-th class. For convenience, the samples of the same class are listed together in a training set, which comprises a matrix, $X\in {ℜ}^{D\times N}$. Given a testing sample, $y\in {ℜ}^{D}$, the SRC aims to solve the following problem:

(3) $$\min_{w}{∥w∥}_{0}\;s.t.\;{∥y-Xw∥}_{2}\leq \varepsilon$$

where ε is a small error constant. $w\in {ℜ}^{N}$ denotes the representation coefficients and ${∥w∥}_{0}$ counts the nonzero entries of w. In general, the problem of (3) can be solved by orthogonal matching pursuit (OMP) [25] to acquire an optimal sparse solution, $w^{*}$. Assuming that $w_{i}^{c}\in w^{*}$ corresponds to the representation coefficient of $x_{i}^{c}$, the reconstruction residual of the *c*-th samples is defined as

(4) $$R_{c}={∥y-\sum_{i=1}^{N_{c}}w_{i}^{c}x_{i}^{c}∥}_{2}.$$

Based on Equation (4), the label of y corresponds to the class with minimum reconstruction residual, i.e., $\min_{c\in \{1,2,\ldots ,C\}}R_{c}$.

SRC has the advantages of high classification accuracy and few tunable hyper-parameters; nevertheless, it is considerably time-consuming because it solves the representation coefficients using all the training samples and it depends on an iteration algorithm to acquire a solution [26].

To alleviate the problems aforementioned, Xu et al. [26] put forward a simple and fast modified SRC. The basic idea of their method is to select only the nearest training sample of the testing sample from each class and then express the testing sample as a linear combination of the selected training samples, where the representation coefficients can be easily solved by the least square algorithm; we call this approach the NSRC approach. After obtaining the representation coefficients, each selected training sample is employed to reconstruct the testing sample, as expressed in Equation (4), and the predict label of the testing sample corresponds to the category with minimum reconstruction residual. In comparison with the SRC, the NSRC represents the testing sample as a linear combination of the selected *C* samples rather than the whole training samples. Additionally, the NSRC uses a least squares approach instead of iterative algorithms, such as OMP. As a result, the NSRC is inherently faster and less computationally demanding than the SRC. However, the NSRC may lead to a loss of classification accuracy because it relies on only one training sample from each class to represent the testing sample, leading to insufficient representation capability and increasing the risk of misclassification.

Aiming to resolve the above issues, this paper develops an improved sparse-representation-based classifier (ISRC), and its basic steps are as follows.

(1) Select the *k*-nearest training samples of the testing sample, y, from every class based on Euclidean distance, which are denoted as $\left\{x_{i}^{c},i=1,2,\ldots ,k;c=1,2,\ldots ,C\right\}$. Here, $x_{i}^{c}$ denotes the *i*-th selected training sample in the *c*-th class.

(2) Use the selected samples to represent y, i.e., $y=\sum_{c=1}^{C}\sum_{i=1}^{k}w_{i}^{c}x_{i}^{c}$, where $w_{i}^{c}$ denotes the representation coefficient corresponding to $x_{i}^{c}$, and these representation coefficients can be solved efficiently through the least squares approach.

(3) Calculate the reconstruction residual of the *c*-th, which is a normalized value:

(5) $$h_{c}=\frac{∥y-\sum_{i=1}^{k}w_{i}^{c}x_{i}^{c}{∥}_{2}}{\sum_{c=1}^{C}{∥y-\sum_{i=1}^{k}w_{i}^{c}x_{i}^{c}∥}_{2}},\;c=1,2,\ldots ,C$$

where $\sum_{c=1}^{C}h_{c}=1$. Here, $h_{c}$ can measure the deviation degree of y from the samples in the *c*-th class; the smaller it is, the more likely y is to be from the corresponding class.

(4) Determine the predict label of y, which is $\min_{c\in \{1,2,\ldots ,C\}}h_{c}$.

Some analyses of the proposed ISRC are presented here. First, the ISRC is viewed as a special case of the SRC. Indeed, if the linear combination of the ISRC is forcibly rewritten as a linear combination of all the training samples, then the representation coefficients for the unselected samples are set to zero. In the ISRC, only kC training samples instead of all training samples are selected to represent the testing sample, and the representation coefficients are solved using the least squares approach rather than the iterative algorithm. Hence, the ISRC is naturally less time-consuming than the SRC, thereby inheriting the computational efficiency advantage of the NSRC. Second, the ISRC is a generalized version of the NSRC, since the former can adjust *k* to pursue an excellent classification performance, while the latter is a fixed classifier with k=1. Thus, with a proper choice of *k*, the ISRC has sufficient capability to represent the test sample, resulting in higher classification accuracy compared to the NSRC.

## 4. The Proposed Ensemble Approach for Fault Diagnosis of Machinery

**Figure 1:**
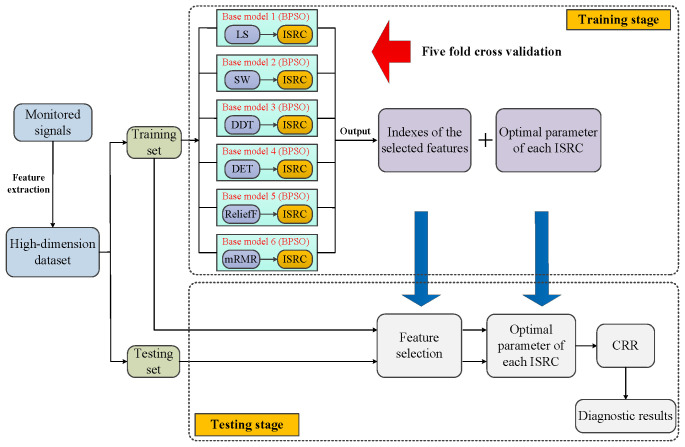
Illustration of the proposed method for fault diagnosis.

Based on six filters, BPSO, and the ISRC, we propose an ensemble approach which is aggregated by six base models. Each base model consists of a filter and the ISRC, numbered according to the order of the filters, as discussed in Section 2.1. The illustration of the proposed method for mechanical fault diagnosis is shown in Figure 1, where the detailed description is presented as follows.

First, the monitored signals of machinery are collected, and these are then processed to extract representative features that are indicative of mechanical health conditions. Here, it is necessary to point out that feature extraction is not the focus of this work; thus, existing advanced feature extraction methods are directly employed. After feature extraction, a high-dimensional mechanical dataset can be obtained, which is then divided into training set and testing set. For the convenience of description, we denote $X=\{x_{i}^{c},i=1,2,\ldots ,N_{c};$$c=1,2,\ldots ,C;\sum_{c=1}^{C}N_{c}=N\}$ as the training set, containing *N* samples coming from *C* health conditions, where $x_{i}^{c}\in {ℜ}^{D}$ refers to the *i*-th sample of the *c*-th class, which has *D* features. $N_{c}$ is the number of samples in the *c*-th class. This is conducted without loss of generality, assuming that $y\in {ℜ}^{D}$ is a testing sample.

Subsequently, the following two stages are carried out sequentially, i.e., the training stage and the testing stage.

### 4.1. Training Stage

This stage only makes use of the training set with the task of determining the indexes of the features to be selected and acquiring the optimal *k* of each ISRC. To achieve this, the following several procedures need to be conducted sequentially.

#### 4.1.1. Feature Sorting

The training set is separately fed into each filter to output the corresponding ranked feature sequence, where the top-ranked features are more significant.

#### 4.1.2. BPSO

After sorting features, the number of the selected features, *r*, remains unknown. Previous studies [27] assigned the same *r* to all filters, potentially resulting in a suboptimal *r* value. Similar to the filter, the ISRC has a tunable parameter, *k*, which it is impractical to fix arbitrarily. Hence, each base model composed of a filter and an ISRC requires careful tuning to determine the optimal *r* and *k*, thereby enhancing the classification performance. Obviously, both the *r* and the *k* are positive integers, and it is not easy to acquire the optimal *r* and *k* simultaneously due to the problem having an NP-hard nature. Additionally, the traditional grid search strategy is impracticable due to the vast search space. Consequently, we turn to BPSO, a swarm intelligence optimization algorithm known for finding global optima. Moreover, the particle in BPSO can effortlessly track integer values for *r* and *k*.

**Figure 2:**
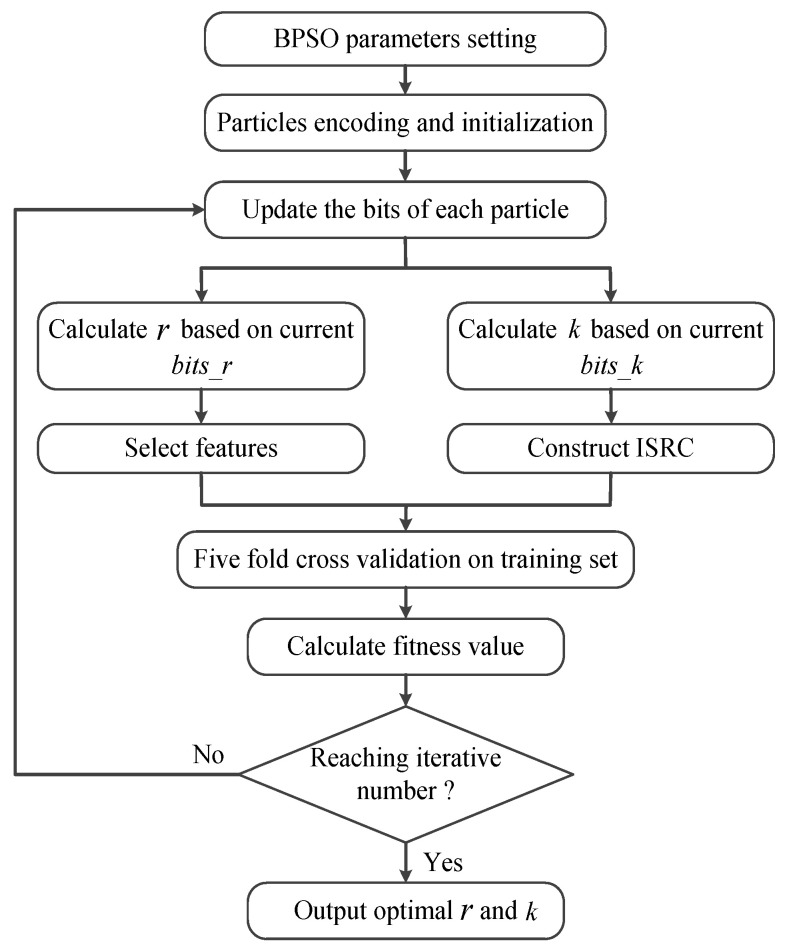
Flowchart of BPSO for each base model.

BPSO is performed on each base model in turn to obtain the optimal *r* and *k* for each base model. The stepwise flowchart of BPSO for each base model is presented in Figure 2, and the specific implementation procedure is available in Algorithm 1.
**Algorithm 1** Optimization procedure of BPSO.**Input:** The iteration number K, the particles number n, the encoded number for each particle d, the training dataset X**Initialization:** position matrix $P\in {ℜ}^{d\times n}$, velocity matrix $V\in {ℜ}^{d\times n}$, The optimal position of each particle $\mathrm{pbest}\in {ℜ}^{d\times n}$, the optimal value of each particle $\mathrm{pbestvalue}\in {ℜ}^{n}$, the global optimal value gbestvalue1:**for** i=1:K** do**2:    **for** j=1:n **do**3:        For $P_{j}$, calculate fitness value in (7) combined with training dataset X4:        Compare fitness value with ${\mathrm{pbestvalue}}_{j}$ and gbestvalue5:        Update ${\mathrm{pbest}}_{j}$ and gbest6:    **end for** Introduce acceleration factors, random numbers, and compute inertia weight7:    **for** j=1:n **do**8:        Update $V_{j}$ as described in (1)9:        Limit $V_{j}$ by means of $V_{j}=1/\left(1+exp\left(V_{j}\right)\right)$10:   Restrict $P_{j}$ via (2)11:    **end for**12:**end for**

*Step 1:* The parameters related to BPSO are set: the acceleration factors, $c_{1}$ and $c_{2}$; the maximum and minimum inertia weights, $w_{max}$ and $w_{min}$; the maximum number of iterations, $n_{iter}$; the particle number, $n_{pop}$; the number of bits, $n_{bits1}$, for calculating *r*; the number of bits, $n_{bits2}$, for calculating *k*. In this paper, $c_{1}=c_{2}=2$, $w_{max}=0.9$, $w_{min}=0.3$, and $n_{iter}=50$. The remaining three parameters will be given in the later experiments.

**Figure 3:**
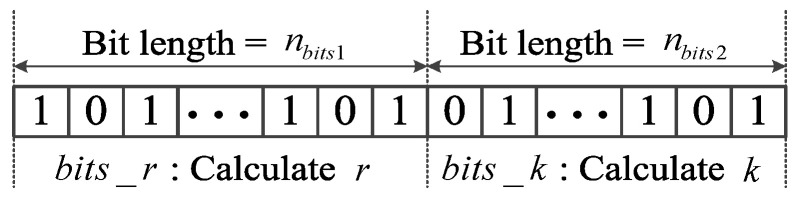
The particle coding of BPSO.

*Step 2:* All particles are encoded with random values of 0 or 1 for initialization. It should be noted that there are a total of $n_{bits1}+n_{bits2}$ bits for coding each particle composed of two parts, as illustrated in Figure 3. The first part (bits_r) and the second part (bits_k) aim to denote the values of *r* and *k*, respectively. Given a code containing *m* bits, the value it denotes is

(6) $$value=\sum_{i=1}^{m}v\left(i\right)2^{m-i}$$

where $v\left(i\right)$ refers to the value of the *i*-th bit, which equals to 0 or 1. According to the definition of Equation (6), the values of *r* and *k* are easily calculated by setting $m=n_{bits1}$ and $m=n_{bits2}$, respectively. Taking bits_r=101011 and bits_k=1100 as examples, the calculated values are r=43 and k=12.

*Step 3:* Here, one starts to carry out the BPSO iteration by updating the bits of each particle. Based on the current particles, the values of *r* and *k* are calculated via Equation (6); then, the corresponding features are selected and the ISRC is constructed. After that, the fitness value of each particle is calculated based on the calculated *r* and *k*, which is acquired by means of five fold cross-validation. To be specific, the whole training set which has selected the corresponding *r* features is divided into five equal folds, of which four folds are used for training and the remaining one is responsible for testing. Each fold is used for testing once, so five classification accuracies can be obtained, which are denoted as $accuracy\left(i\right)\in \left[0,1\right],$ i=1,2,3,4,5. Subsequently, the fitness value is defined as

(7) $$fitness\;value=-\frac{1}{5}\sum_{i=1}^{5}accuracy\left(i\right)+0.001r$$

where the first term denotes the average classification accuracy of fivefold cross-validation on the training set. The second term acts as a penalty, which holds that, if two particles achieve the same average classification accuracies, then the particle with a smaller *r* is preferred. Here, an extreme situation is that r=0 or k=0, and we assume that the fitness value of such cases is positive infinite.

*Step 4:* Judge whether the number of iterations reaches the maximum value, $n_{iter}$. If so, then the obtained optimal *r* and *k* are output; otherwise, *Step 3* is repeated to continue the iteration.

Through the process in Figure 2, each base model can obtain its optimal *r* and *k* independently. After training, the values of *k* in six ISRCs together with six groups of the indexes of the features selected by six filters are stored for the use of the subsequent testing stage.

### 4.2. Testing Stage

This stage works by inputting the training set X and the testing sample y into each base model to obtain six single outputs. Then, six single outputs are aggregated to acquire the ensemble output by means of certain aggregation strategy. The detailed steps are as follows.

#### 4.2.1. Output of Each Base Model

Taking base model 1 as an example, the training set X and testing sample y all conduct feature selection based on the indexes determined by LS. After feature selection, both the training set and the testing sample are fed into the ISRC with the optimal *k*; the four steps of the ISRC in Section 3 are carried out sequentially. Finally, the predicted label of the testing sample y can be acquired, which corresponds to its health condition. Simultaneously, the reconstruction residual vector defined in Equation (5) is also obtained, which is denoted as $h_{1}=\left(h_{11},h_{12},\ldots ,h_{1C}\right)\in {ℜ}^{C}$, where $h_{1c}$ refers to the reconstruction residual corresponding to the *c*-th class for c=1,2,…,C. Similarly, the remaining five base models generate the predicted labels of y along with their respective reconstruction residual vectors, which are separately denoted as $h_{2}$, $h_{3}$, $h_{4}$, $h_{5}$, and $h_{6}$.

#### 4.2.2. Aggregation

Six predict labels are obtained for y, which must be further processed to obtain the ultimate output via the aggregation strategy. A commonly used approach is the simple voting approach, as explored in [27]. Nevertheless, the voting strategy may suffer from three weaknesses, as follows. First, the voting strategy fails to provide an accurate judgment of when two classes receive the same maximum number of votes. Second, there may be two similarly small reconstruction residuals in $h_{i}$ for i=1,2,…,6, which obliterates the difference between such two classes. If we arbitrarily determine the predicted label of the *i*-th base model based on these two values, the results may become considerably biased. Third, the voting strategy relies on most base models performing well to achieve an optimal result. Nevertheless, this requirement can not be always ensured.

To overcome these weaknesses, this work proposes an alternative strategy named the cumulative reconstruction residual (CRR) strategy, which is inspired by cumulative probability as discussed in [14]. The rationale of CRR is fairly simple; it works by determining the category of the testing sample, y, as the health condition with the minimum cumulative reconstruction residual. Mathematically, we denote $H=\sum_{i=1}^{6}h_{i}={\left(H_{1},H_{2},\ldots ,H_{C}\right)}^{T}$ as the cumulative reconstruction residual vector, where $H_{c}$ is the cumulative reconstruction residual of the *c*-th class for c=1,2,…,C. The label of y is predicted as $\min_{c\in \{1,2,\ldots ,C\}}H_{c}$. In comparison to voting, CRR offers three significant advantages. First, since CRR makes a decision for y based on the calculated h, it effectively avoids the confused situation that two classes acquire the same maximum votes number, while such a situation is common when undertaking the voting strategy. Second, CRR assesses the overall cumulative reconstruction residual performance across all base models, thereby exacerbating the differences between the classes and enabling explicit and accurate predictions to be made. Third, CRR presents the ensemble result based on the cumulative value of the reconstruction residual, making it more inclusive than voting, as it can accommodate the poorer performances of base models in certain cases.

## 5. Experimental Verification

### 5.1. Experimental Datasets

In this work, six mechanical datasets from four publicly available data repositories were collected to verify the effectiveness of the proposed method. These datasets are diverse and comprehensive, as they include the most widely studied mechanical components, e.g., bearings and gears, constant and variable operation conditions, and single and mixed types of faults. Table 1 summarizes these datasets and their details are described in the following subsections.

#### 5.1.1. CWRU-A and CWRU-B

The CWRU-A and CWRU-B are two datasets about bearings from the Case Western Reserve University (CWRU) Lab [28], and they were collected at sampling frequencies of 12 KHz and 48 KHz, respectively. Both CWRU-A and CWRU-B contain ten health conditions: normal (N), ball fault with 0.007 inches (B007), ball fault with 0.014 inches (B014), ball fault with 0.021 inches (B021), inner race fault with 0.007 inches (IR007), inner race fault with 0.014 inches (IR014), inner race fault with 0.021 inches (IR021), outer race fault with 0.007 inches (OR007), outer race fault with 0.014 inches (OR014), and outer race fault with 0.021 inches (OR021). The vibration signals on the drive end were collected under 1772 rmp (load of 1 hp). Both datasets have 70 training samples and 30 testing samples in each health condition, while each sample contains 1200 data points for CWRU-A and 2400 data points for CWRU-B.

For CWRU-A and CWRU-B, we first decompose each sample into several intrinsic mode functions (IMFs) using empirical mode decomposition. Then, the first five IMFs are utilized and each IMF is processed to extract sixteen statistical features which are from [7], including twenty time domain and four frequency domain features. Afterwards, the features of all IMFs are concatenated to compose a long feature vector containing 80 (16 × 5) features. In this way, each sample is finally represented by an 80-dimensional vector.

#### 5.1.2. MFPT

The MFPT dataset on bearings is provided by Machinery Failure Prevention Technology (MFPT) [29]; it includes data on three health conditions: normal (Normal), inner race fault (IRF), and outer race fault (ORF). The vibration signals of the Normal condition are collected under a load of 270 lbs at a sampling rate of 97,656 Hz, and this condition consists of 286 samples with 2048 data points for each sample. The vibration signals of the IRF and the ORF are collected at a sampling rate of 48,828 Hz under seven operation conditions: 0, 50, 100, 150, 200, 250, and 300 lbs for the IRF and 25, 50, 100, 150, 200, 250, and 300 lbs for the ORF. For both the IRF and the ORF, there are 71 samples in each operation condition, with 2048 data points for each sample. In total, there are 286, 497, and 497 samples in the Normal, IRF, and ORF conditions, respectively. All the samples in each health condition are divided equally into two parts, where the first half and the second half are responsible for training and testing, respectively.

For the MFPT dataset, we take advantage of the feature extraction method presented in [5] to extract three kinds of features based on time domain analysis, empirical mode decomposition, and wavelet packet transform. More concretely, each vibration signal is first processed to calculate six dimensionless time domain parameters (*skewness*, *kurtosis*, *crest indicator*, *clearance indicator*, *shape indicator*, and *impulse indicator* ). Then, the first six IMFs, decomposed through empirical mode decomposition, are described by the above six parameters, respectively. After that, each vibration signal is processed by WPT at a decomposition level of 3 using ’db5’, and the above six parameters are again calculated from each frequency band signal. Lastly, a total of 90 $\left(6+6\times 6+6\times 2^{3}\right)$ features are extracted to represent each sample.

#### 5.1.3. UoC

This dataset comes from the University of Connecticut (UoC) [30] and has been utilized in [31]. The dataset accounts for nine kinds of gear faults: healthy (H), missing tooth (MT), root crack (RC), spalling (S), and chipping tip with five different levels of severity (CT1-CT5). For each health condition, the time domain vibration signals are collected at a sampling frequency of 20 KHz. Each health condition has 70 training samples and 34 testing samples, and each sample has 3600 data points. Similar to the MFPT dataset, we extract 90 features for each sample of the UoC dataset, using the method presented in [5].

#### 5.1.4. SEU-A and SEU-B

The SEU-A and SEU-B datasets are provided by Southeast University (SEU) [32] and can be downloaded from [33]. Two different operation conditions are investigated with rotating speed system loads of 20 HZ-0V and 30 HZ-2V, which correspond to the SEU-A and SEU-B datasets, respectively. Similar to [32], the SEU-A and SEU-B datasets are both mixture datasets, comprising data on healthy state (H), four gear faults (chipped (GC), missing (GM), root crack (GRC), and surface wear (GSW)), and four bearing faults (ball crack (BBC), inner race crack (BIRC), outer race crack (BORC), and a combination both inner and outer race (BIORC) faults). Each health condition contains 150 training and 90 testing samples with 4369 data points for each sample.

For these two datasets, we make use of frequency spectrum partition summation (FSPS) [34] features to represent each sample due to its significant effect. The basic idea behind FSPS is as follows. Given a vibration signal, $f={\left\{f_{i}\right\}}_{i=1}^{I}$, with *I* data points, $s\left(j\right)$ refers to its amplitude frequency spectrum obtained through Fast Fourier Transformation (FFT) for j=1,2,…,J. Then, the feature $X_{FSPS}\left(l\right)$ of the FSPS is calculated by $X_{FSPS}\left(l\right)=\sum_{j=\frac{L+J\left(l-1\right)}{L}}^{\frac{Jl}{L}}s\left(j\right)$, where l=1,2,…,L. For the SEU-A and SEU-B datasets, the vibration signal, which has 4369 data points, contains 4369 amplitude frequency spectra after FFT. Then, 2184 (form 2 to 2185) frequency spectra in total are chosen owing to symmetry. We take *L* as 91 for these two datasets; thus, 91 FSPS features are extracted to describe each sample.

### 5.2. Results of BPSO

**Figure 4:**
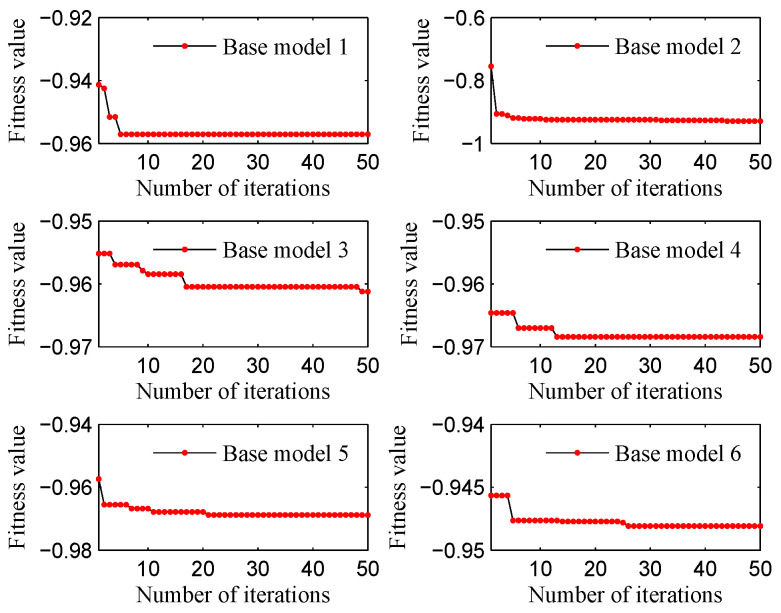
Iterative curves on SEU-B dataset.

First, each base model is optimized independently. To be specific, the filter in each base model takes the training samples as input and produces a ranked feature sequence as output. Then, BPSO is utilized to identify the optimal *r* for the filter and the optimal *k* for the ISRC, respectively. The detailed parameter settings for BPSO are listed in Table 2. BPSO follows the procedure of Algorithm 1 to update the particles; this is conducted with the aim of reducing the fitness value until the stopping criterion $n_{iter}$ is reached. Due to limited space, we present the iterative curves of six base models on the last dataset (i.e., SEU-B), as shown in Figure 4. Finally, the optimal *r* and *k* for each base model across each dataset are recorded in Table 3.

From Table 3, we can see that averages of approximately 43.50% (34.8/80), 37.25% (29.8/80), 21.11% (19/90), 39.11% (35.2/90), 32.75% (29.8/91), and 38.24% (34.8/91) of features are selected for fault classification in the CWRU-A, CWRU-B, MFPT, UoC, SEU-A, and SEU-B datasets, respectively. This indicates that not all the features are beneficial for the fault diagnosis process, and feature selection is necessary.

### 5.3. Diagnostic Results and Analysis

The testing accuracies of each base model at the corresponding optimal *r* and *k*, listed in Table 3, are summarized in the first six rows of Table 4. Meanwhile, the outputs of these six base models are aggregated by CRR to acquire the testing accuracies of the proposed method, which are listed in the last row of Table 4. For the sake of better comparison, four other ensemble methods are investigated, which are given different *r* or *k* while all of them utilize CRR for aggregation. We denote $\bar{r}$ and $\bar{k}$ as the average value of *r* and *k*, which have been recorded in last column of Table 3. Detailed descriptions of these ensemble methods are provided here.

- ***Ensemble method 1***: All the base models take $\bar{r}$ for each filter, while they still take the optimal *k* for the ISRC.
- ***Ensemble method 2***: All the base models take $\bar{k}$ for each ISRC, while they still take the optimal *r* for the filter.
- ***Ensemble method 3***: All the base models take $\bar{r}$ and $\bar{k}$ for each filter and each ISRC.
- ***Ensemble method 4***: All the base models take the optimal *k* for the ISRC, whereas they take the maximum value of *r* for each filter. This refers to the fact that feature selection is not carried out in each base model.

The testing accuracies of the ensemble methods 1–4 are tabulated in the seventh–tenth rows of Table 4, respectively.

From Table 4, we can see that the proposed method achieves the best result on each dataset consistently, which demonstrates its superiority in fault diagnosis for machinery. Moreover, it is noticed that the testing accuracy of base model 1 is satisfactory on the CWRU-A dataset, whereas it is relatively poor on the UoC dataset. Base model 3 performs well on the UoC, SEU-A, and SEU-B datasets, but under-performs on the MFPT dataset. These observations indicate that the single model (only using a single filter and the classifier) tend to produce inconsistent results across the different datasets. Among the four ensemble methods, it seems that ensemble methods 1–3 outperform all base models but fall short of the proposed method, and ensemble method 4 produces relatively poor diagnostic results.

Overall, the experimental results in Table 4 demonstrate three key findings: (1) Feature selection is immensely necessary as it holds the potential to improve diagnostic accuracy by selecting a subset of salient features. (2) Combining the feature subsets selected by multiple filters yields better results than using a single feature subset. (3) Assigning optimal r and k values to each base model in the ensemble approach is more effective than having all models share the same user-defined values.

### 5.4. Comparison with Other Classifiers

**Figure 5:**
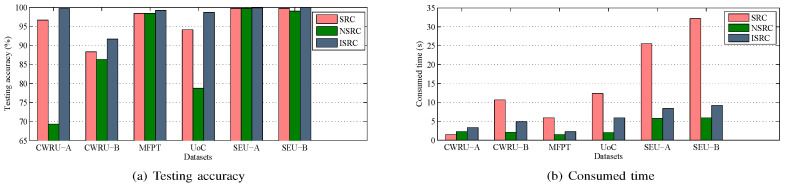
Comparison results of SRC, NSRC, and ISRC in terms of the testing accuracy and the consumed time.

The ISRC is proposed as one of the main contributions of this paper for fault classification. To verify its effectiveness, this subsection compares the ISRC with its two related versions, i.e., the SRC and the NSRC. For fair comparison, all the base models are configured with the SRC and the NSRC while maintaining the optimal r values in an unchanged state. In this case, all three classifiers are fed with the same set of features. In the evaluation, two metrics are considered, i.e., testing accuracy and consumed time. Here, it should be noted that the consumed time refers to the sum of the time spent by the classifiers in the six base models. The testing accuracies and the consumed time for different datasets are displayed in Figure 5. Here, it is noted that the experiments were carried out in the MATLAB 2012a environment with an Intel Core i5-4460 3.2 GHz( Intel Corporation, Santa Clara, California, United States.) processor and 8 GB of main memory.

From Figure 5, we can observe that the ISRC achieves the highest testing accuracy on each dataset. The time it takes is slightly longer than that taken by the NSRC but significantly shorter than that taken by the SRC on all datasets except the CWRU-A dataset. Moreover, the SRC achieves slightly lower testing accuracies than the ISRC. nevertheless, it takes much more time in most datasets, limiting its practical applications. Although the NSRC achieves the fastest performance among the three classifiers, its testing accuracy is unsatisfactory, particularly on the CWRU-A and UoC datasets. By comparison, the proposed ISRC is a promising classifier, achieving high classification accuracy with significantly reduced time consumption.

### 5.5. Comparison with Voting Strategy

**Figure 6:**
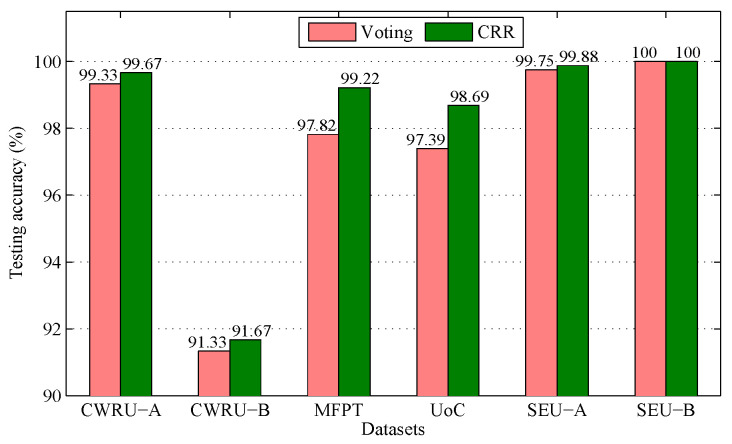
Comparison between voting and CRR.

Recall that this work devises a new aggregation strategy called CRR, which takes the place of the widely used voting strategy. To verify its effectiveness, we compare the testing accuracies of the methods which separately adopt CRR and voting on different datasets, as depicted in Figure 6.

From Figure 6, one can observe that the CRR-based method is superior to the voting-based method in terms of testing accuracy for five out of six datasets, while they acquire the same result on the sixth dataset. These results demonstrate that the CRR-based method is superior to the voting-based method, and CRR is more suitable for aggregation than voting.

**Figure 7:**
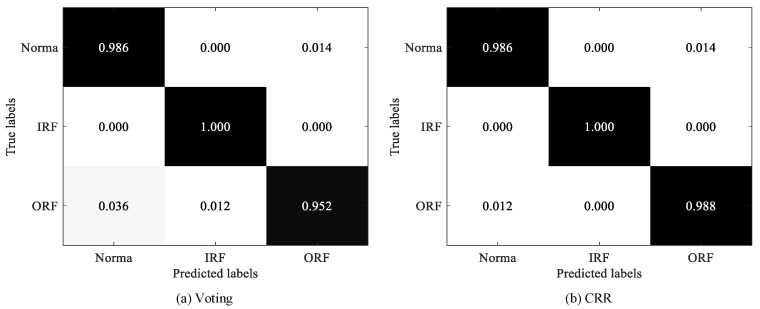
Confusion matrices of voting and CRR on MFPT dataset.

**Figure 8:**
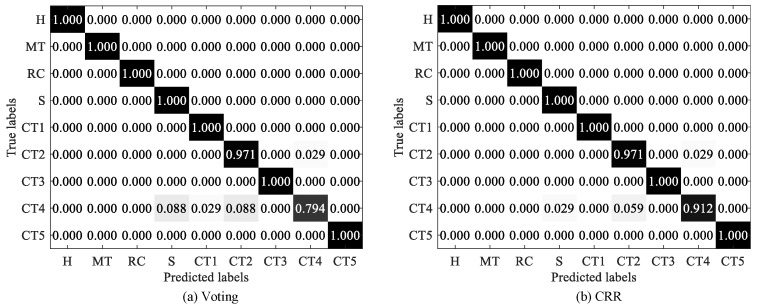
Confusion matrices of voting and CRR on UoC dataset.

In addition, Figure 6 shows that CRR performs significantly better than voting on the MFPT and UoC datasets, where the accuracies of CRR are 1.4 % and 1.3 % higher than voting on MFPT and UoC, respectively. To make a more detailed comparison, we display the confusion matrices of two strategies on the two datasets, as shown in Figure 7 and Figure 8. As exhibited in Figure 7, both voting and CRR perform equally well in diagnosing Normal faults and IRFs, but the latter significantly outperforms the former in diagnosing ORFs (i.e., outer race fault). As exhibited in Figure 8, CRR performs significantly better than voting in diagnosing CT4 (i.e., chipping tip with several level).

To further investigate the way in which CRR is superior to voting strategy, we revisit the CWRU-A, CWRU-B, MFPT, UoC, and SEU-A datasets. Specifically, we concentrate on studying the special testing samples in these datasets, which were correctly classified by the CRR-based method but were mistakenly classified by the voting-based method. For each special testing sample, we observe its reconstruction residual vector, $h_{i}$, obtained by the *i*-th base model and the cumulative reconstruction residual vector, H, obtained by CRR. By analysis, the following three reasons may make CRR superior to voting.

First, the voting-based approach struggles to make decisions when two classes acquire the same maximum number of votes; CRR overcomes this limitation. Such a situation appears in the CWRU-B, MFPT, and UoC datasets, as shown in Table 5, Table 6 and Table 7, respectively. It is noted that both IR007 and OR021 in Table 5 receive the greatest votes, i.e., 3; both Normal faults and ORFs in Table 6 receive the greatest votes, i.e., 3; both S and CT2 in Table 7 receive the greatest votes, i.e., 2. In these cases, the voting-based method leads to random and ambiguous decisions. On the contrary, the proposed CRR method accumulates the reconstruction residuals for each class, enabling accurate decisions.

Second, the voting-based approach is likely to make an opposite decision when two classes are extremely similar. Such a situation appears in the CWRU-A dataset, as shown in Table 8. Here, the voting-based approach easily makes a decision for this testing sample because the class with most votes, which is OR014, is unique. Unfortunately, such a result is incorrect. With careful observation of Table 8, we can see that OR014 and B021 are easily confused because their reconstruction residual values are extremely similar in some base models, such as base models 1–5. After culminating through CRR, the differences between the classes are slightly amplified, allowing for correct predictions.

Third, the voting-based approach requires that most base models work well, such that the perfect result could be obtained; meanwhile, CRR is more lenient. This situation is evident in the SEU-A dataset, as shown in Table 9. In this table, four base models incorrectly classify this testing sample, while only two base models classify it correctly. Clearly, the voting-based method incorrectly classifies this sample to the health condition of GC, whereas its true condition is BBC, which is correctly classified by the CRR-based method. Observations reveal that the reconstruction residual difference between GC and BBC is considerably small for base model 2, 4, and 6 but very big for base models 3 and 5, which means that base models 3 and 5 are dominant base models with the capability to provide a correct decision. In other words, even when most base models perform poorly, the ensemble performance can still be satisfactory. This standpoint also validates the idea that CRR is more inclusive than voting.

## 6. Conclusions

This paper proposes an ensemble approach for intelligent fault diagnosis of machinery, which consists of several filters and classifiers. To overcome the limitations of the SRC, we propose an improved variant of the SRC, namely the ISRC, for fault classification. Six filters are utilized, with each filter combined with the ISRC to constitute a base model. For each base model, the filter selects a subset of features for training and testing the ISRC; here, two hyper-parameters involved in the filter and the ISRC approaches are optimized by the BPSO algorithm. The outputs of all the base models are combined by CRR, replacing the commonly used voting strategy. Furthermore, six datasets related to bearings and gears are utilized to evaluate the performance of the proposed approach. Through several experiments, the following conclusions are drawn:

(1) Feature selection is significantly essential, as it enhances diagnostic accuracy by selecting the most discriminative features.

(2) Combining multiple filters and classifiers for fault diagnosis is more effective than using a single filter and a single classifier, which can reduce the variability in the results over different datasets.

(3) Two hyper-parameters involved in the filter and the classifier of each base model are supposed to be optimized instead of manually assigning the same value, promoting higher diagnostic accuracy.

(4) In comparison to the SRC, the developed ISRC not only inherits its merit of high classification accuracy, it also overcomes its drawback of high computational time. Thereby, the ISRC emerges as a promising classifier.

(5) The designed CRR outperforms the voting approach, as it effectively addresses three limitations of the voting strategy. Therefore, CRR is more suitable for aggregation.

In this work, we focus on developing a BPSO-optimized ensemble approach to achieve a high diagnosis performance, and the method is simply evaluated using six regular datasets, where sufficient and identical samples are provided for the normal condition and each faulty condition. Nevertheless, very few, if any, faulty examples (labeled and unlabeled) are available due to safety considerations [35]. Few or even zero-fault-shot learning is a current research hotspot, and is of great and practical significance in mechanical fault diagnosis. Therefore, we will explore in this area in the future.

## Figures and Tables

**Table 1:** Description of experimental datasets.

Name	Object	Number of Features	Number of Health Conditions
CWRU_A	Bearing	80	10
CWRU_B	Bearing	80	10
MFPT	Bearing	90	3
UoC	Gear	90	9
SEU_A	Gear + Bearing	91	9
SEU_B	Gear + Bearing	91	9

**Table 2:** Parameter settings in BPSO.

	CWRU-A	CWRU-B	MFPT	UoC	SEU-A	SEU-B
$n_{bit1}$	6	6	6	6	6	6
$n_{bit2}$	4	5	5	5	4	4
$n_{pop}$	15	15	15	15	10	10

**Table 3:** The optimal *r* and *k* obtained by BPSO.

Dataset	Parameter	Base Model						Average
		**1**	**2**	**3**	**4**	**5**	**6**	
CWRU-A	*r*	42	53	26	26	29	33	34.8
	*k*	15	15	11	14	15	15	14.2
CWRU-B	*r*	34	35	32	31	19	28	29.8
	*k*	25	21	30	17	11	17	20.2
MFPT	*r*	19	24	14	15	22	20	19.0
	*k*	20	30	24	26	18	31	24.8
UoC	*r*	43	47	33	17	32	39	35.2
	*k*	26	31	27	20	28	27	26.5
SEU-A	*r*	20	40	26	17	26	50	29.8
	*k*	15	15	13	12	8	15	13.0
SEU-B	*r*	31	51	27	28	23	49	34.8
	*k*	15	15	15	12	14	15	14.3

**Table 4:** Testing accuracies on different machinery datasets (%).

Methods	CWRU-A	CWRU-B	MFPT	UoC	SEU-A	SEU-B	Average
Base model 1	99.33	90.67	98.44	95.42	98.52	98.64	96.84
Base model 2	97.33	86.33	95.01	94.44	96.91	97.16	94.53
Base model 3	98.33	90.00	95.48	97.39	99.14	99.26	96.60
Base model 4	98.67	90.33	95.63	93.46	98.40	99.01	95.92
Base model 5	98.67	85.33	98.28	97.06	99.01	99.26	96.27
Base model 6	98.67	87.33	98.13	95.75	99.01	99.26	96.36
Ensemble method 1	99.33	90.00	99.06	**98.69**	**99.88**	**100.00**	97.83
Ensemble method 2	**99.67**	90.33	98.91	98.37	**99.88**	**100.00**	97.86
Ensemble method 3	99.33	89.67	99.06	98.69	99.75	**100.00**	97.75
Ensemble method 4	94.67	90.33	93.76	98.04	98.02	97.90	95.45
Proposed method	**99.67**	**91.67**	**99.22**	**98.69**	**99.88**	**100.00**	**98.19**

**Table 5:** Reconstruction residual values of one special testing sample on CWRU-B.

Health Conditions	Label	Base Model 1	Base Model 2	Base Model 3	Base Model 4	Base Model 5	Base Model 6	Proposed
N	1	0.0977	0.1080	0.1025	0.1038	0.1060	0.1017	0.6196
B007	2	0.0941	0.1068	0.1029	0.0885	0.0961	0.0947	0.5830
B014	3	0.1073	0.0957	0.1030	0.1052	0.0832	0.1024	0.5968
B021	4	0.0963	0.1058	0.0951	0.0991	0.1045	0.0967	0.5975
IR007	5	**0.0769**	0.0866	0.0871	**0.0804**	0.0909	**0.0824**	0.5042
IR014	6	0.1112	0.1075	0.1100	0.1100	0.1167	0.1090	0.6645
IR021	7	0.1062	0.1077	0.1070	0.1052	0.1126	0.1062	0.6450
OR007	8	0.1154	0.1088	0.1113	0.1063	0.1127	0.1154	0.6700
OR014	9	0.1000	0.1078	0.0988	0.1109	0.1048	0.1009	0.6231
OR021	10	0.0950	**0.0653**	**0.0823**	0.0906	**0.0726**	0.0906	**0.4964**
Predict label		5	10	10	5	10	5	10

**Table 6:** Reconstruction residual values of one special testing sample on MFPT.

Health Conditions	Label	Base Model 1	Base Model 2	Base Model 3	Base Model 4	Base Model 5	Base Model 6	Proposed
Normal	1	0.2469	0.3775	0.2616	0.2416	0.4402	0.3280	1.8957
IRF	2	0.4022	0.4585	0.4227	0.4239	0.4492	0.4490	2.6056
ORF	3	0.3508	0.1640	0.3157	0.3345	0.1107	0.2230	1.4987
Predict label		1	3	1	1	3	3	3

**Table 7:** Reconstruction residual values of one special testing sample on UoC.

Health Conditions	Label	Base Model 1	Base Model 2	Base Model 3	Base Model 4	Base Model 5	Base Model 6	Proposed
H	1	0.1383	0.1171	0.1193	0.1192	0.1264	0.1115	0.7318
MT	2	0.1614	0.1418	0.1463	0.1549	0.1486	0.1491	0.9020
RC	3	**0.0803**	0.0828	0.0925	0.0925	0.0861	0.0710	0.5051
S	4	0.1028	0.1137	0.0782	**0.0637**	**0.0625**	0.1065	0.5274
CT1	5	0.1026	0.1004	0.1260	0.1266	0.1266	0.1149	0.6971
CT2	6	0.0887	0.1011	**0.0544**	0.0640	0.0626	**0.0644**	**0.4352**
CT3	7	0.1396	0.1474	0.1371	0.1354	0.1315	0.1503	0.8412
CT4	8	0.0846	**0.0533**	0.1254	0.1345	0.1361	0.1052	0.6390
CT5	9	0.1018	0.1426	0.1208	0.1093	0.1195	0.1272	0.7211
Predict label		3	8	6	4	4	6	6

**Table 8:** Reconstruction residual values of one special testing sample on CWRU-A.

Health Conditions	Label	Base Model 1	Base Model 2	Base Model 3	Base Model 4	Base Model 5	Base Model 6	Proposed
N	1	0.1092	0.1075	0.1103	0.1099	0.1103	0.1104	0.6575
B007	2	0.0964	0.0960	0.0981	0.0961	0.0956	0.0968	0.5790
B014	3	0.1164	0.1249	0.1007	0.1073	0.1093	0.1091	0.6676
B021	4	**0.0683**	0.0711	0.0754	0.0769	0.0724	**0.0484**	**0.4125**
IR007	5	0.1081	0.1134	0.1082	0.1081	0.1078	0.1110	0.6565
IR014	6	0.1002	0.0896	0.1025	0.0959	0.0972	0.1075	0.5930
IR021	7	0.1050	0.1059	0.1107	0.1081	0.1091	0.1073	0.6461
OR007	8	0.1145	0.1143	0.1140	0.1180	0.1183	0.1161	0.6952
OR014	9	0.0690	**0.0673**	**0.0650**	**0.0654**	**0.0666**	0.0828	0.4160
OR021	10	0.1129	0.1100	0.1151	0.1142	0.1136	0.1107	0.6765
Predict label		4	9	9	9	9	4	4

**Table 9:** Reconstruction residual values of one special testing sample on SEU-A.

Health Conditions	Label	Base Model 1	Base Model 2	Base Model 3	Base Model 4	Base Model 5	Base Model 6	Proposed
H	1	0.1935	0.2034	0.1280	0.1855	0.1323	0.1344	0.9772
GC	2	0.0441	**0.0362**	0.1380	**0.0394**	0.1418	**0.0275**	0.4270
GM	3	0.1404	0.1985	0.1232	0.1471	0.1034	0.1870	0.8996
GRC	4	0.1810	0.1105	0.1257	0.1656	0.1042	0.1341	0.8213
GSW	5	0.0929	0.0542	0.1247	0.1058	0.1173	0.1184	0.6132
BBC	6	0.0685	0.1057	**0.0161**	0.0577	**0.0209**	0.0761	**0.3450**
BIRC	7	**0.0336**	0.0863	0.1128	0.0462	0.1288	0.0984	0.5061
BORC	8	0.0761	0.0453	0.1082	0.0838	0.1288	0.0890	0.5312
BIORC	9	0.1698	0.1600	0.1232	0.1688	0.1225	0.1350	0.8794
Predict label		7	2	6	2	6	2	6

## Data Availability

Dataset available on request from the authors.
